# The Impact of Living in a Care Home on the Health and Wellbeing of Spinal Cord Injured People

**DOI:** 10.3390/ijerph120404185

**Published:** 2015-04-15

**Authors:** Brett Smith, Nick Caddick

**Affiliations:** Peter Harrison Centre for Disability Sport, Loughborough University, Loughborough, Leciestershire, LE11 3TU, UK; E-Mail: N.D.Caddick@lboro.ac.uk

**Keywords:** spinal cord injury, care home, quality of life, health, wellbeing

## Abstract

In the UK, 20% of people with spinal cord injury (SCI) are discharged from rehabilitation into an elderly care home. Despite this, and knowledge that the home is central to health and wellbeing, little research has examined the impact of being in care homes on the health and wellbeing of people with SCI. The purpose of this study was to address this gap. Twenty adults who lived in care homes or had done so recently for over two years were interviewed in-depth. Qualitative data were analyzed using inductive thematic analysis. Analyses revealed that living in a care home environment severely damages quality of life, physical health and psychological wellbeing in the short and long-term. Reasons why quality of life, health, and wellbeing were damaged are identified. These included a lack of freedom, control, and flexibility, inability to participate in community life, inability to sustain relationships, safety problems, restricted participation in work and leisure time physical activity, lack of meaning, self-expression, and a future, loneliness, difficulties with the re-housing process, depression, and suicidal thoughts and actions. It is concluded that for people with SCI, the care home environment violates social dignity, is oppressive, and denies human rights. Implications for housing and health care policies are also offered.

## 1. Introduction

Spinal cord injury (SCI), as a major disruptive life event, instigates a multiplicity of complex issues that the newly disabled person, his or her family, and public policy need to deal with. One fundamental issue involves housing [1]. Following a lengthy period of rehabilitation in a spinal injury hospital, the individual with SCI is discharged into the community. Once in the community they require an accessible house in order to meet their new needs. However, for many disabled people such housing is scarce [1]. As a result of this scarcity, often the only place to live for a significant amount of people with SCI is in a care home—a type of residential accommodation, sometimes also referred to as a nursing home, where a number of elderly people often live and on-site care services are provided. 

However, what impact does living in a care home have on the health and wellbeing of people with SCI? This question is of major importance for several interrelated reasons. In the UK, where this study was conducted, every eight hours someone is paralyzed by a SCI [2]. It is estimated that there are now over 40,000 people with SCI living in the UK [2]. What is more, 20% of people with SCI are discharged from rehabilitation into care homes designed for the elderly. Rather than being a temporary or short-term housing solution, it is common for a person with SCI to live permanently in a care home for over 2 years before an alternative home is found. Living in this institutional environment also represents a major financial issue for the UK health and public policy system. As in many other countries (e.g., Australia and Canada), it is the local public authority that funds the huge economic costs that go with living in a care home. It is important moreover to examine the impact of living in a care home on people’s health and wellbeing because this kind of housing is much more than a physical dwelling, composed of simply bricks and mortar. It can be conceptualized as a home environment that is central to health and wellbeing [3,4]. That is to say, the institutional care home is a relational space where interactions, material social practices, and experiences occur to give shape and meaning to people’s everyday lives. In this regard, home environments, like a care home, can potentially impact significantly on the health and wellbeing of all individuals. 

Despite the importance of understanding the impact of living in a care home environment on the health and wellbeing of people with SCI, there is a lack of research on this topic. This paper addresses this significant knowledge gap. However, this is not to suggest that there is a complete absence of empirical studies on disability and housing. For example, social scientific work has been conducted on disability, embodiment and the meaning of home [5] as well as the choices, opportunities and barriers to housing for disabled people in general [1]. Research has also examined what constitutes an adequate home environment for adults with mobility disabilities, such as muscular dystrophy [4]. Using a critical disability ethics approach, this research revealed that what constitutes an adequate home environment was one that enables and promotes social dignity by providing access to seven essential conditions. These conditions considered necessary for a dignity-enabling home were: the ability to form and sustain meaningful relationships; access to community and civic life; access to control and flexibility of daily activities; access to opportunities for self-expression and identity affirmation; access to respectful relationships with attendants; access to opportunities to participate in school, work or leisure; and access to physical, psychological and ontological security [4]. 

Notwithstanding such research, it remains that there is a lack of research that has focused on the impact of living in a care home environment on the health and wellbeing of people with SCI. The purpose of this research was to examine this impact. To do this, qualitative methods were used.

## 2. Methods 

### 2.1. Philosophical Assumptions and Data Collection Methods

The design of this qualitative study is underpinned by ontological relativism (*i.e.*, reality is multiple, created, and mind-dependent) and epistemological constructionism (*i.e.*, knowledge is constructed and subjective) [6]. Qualitative research is different (not better or worse) to quantitative research and was chosen for several reasons. Rather than providing superficial accounts of people’s lives or reducing humans to a number, it is faithful to “real-life” phenomena in all of their richness and to the realities of people as *human*-beings [6,7]. Instead of imposing on people and limiting what they can express, qualitative methods also enable people to communicate—in their own words and on their own terms—what certain phenomena mean to them in complex ways [7,8].

After gaining university ethical approval for the study, a criterion-based purposive sampling strategy was used to recruit participants [6]. In this kind of sampling participants are chosen because they share a particular attribute or characteristic (e.g., SCI) and experience (e.g., living in a care home). Thus, criterion-based purposive sampling was deemed an appropriate strategy to use. That is, the rationale for criterion-based purposive sampling lies in its ability to provide relevant and rich information that will help understand the research problem and purpose. The criteria that guided inclusion in the sampling process were people who: (a) had sustained a traumatic SCI and are registered as disabled by a UK Government authority (e.g., employment or housing); (b) are aged 18 years or over; and (c) currently live in a care home or had lived in one within the last 6 months. The latter group of people who had recently left a care home were included as they could uniquely provide deep insight into living in a care home from a position of temporal distance. Such distance can allow people to reflexively share information that they might not have thought useful when living in the care home itself [7,8]. To recruit a sample, an open letter was placed in U.K. disability newsletters/Internet sites, inviting people who met the sampling criteria to take part in the study. Recruitment of participants continued until data saturation was achieved. Data saturation refers to an iterative process that involves collecting and transcribing initial data, immediately assessing it, and then continuing to collect and assess data until nothing new is generated [8,9]. Data saturation is achieved when there are no more emergent patterns/themes in the data; data begins to repeat itself [8,9]. The result was a recruited sample of 20 people. Specifically, 15 males and 5 females aged between 21 and 70 years (mean = 31 years of age) and who had, on average, lived in care home for 2.3 years were recruited. The preponderance of males reflects the fact that the vast majority of spinal injuries happen to young men. Fourteen people lived in a care home and 6 lived in the community, having recently left a care home. 

All participants were involved in a semi-structured life story interview. Each interview was recorded and lasted on average 2 hours. In each interview, the interviewer invited each participant to tell stories about their own life and how it had been lived over time within the care home [6,7,8]. An interview guide was used to help facilitate discussion. Questions included in the guide were, “Can you tell me about your life in the care home?”, “Can you describe how it feels to be living in a care home?”, “What impact does living in a care home have on your life?”, and “Do you have hopes about your future?” Clarification, elaboration, and detail orientated probes, that is, curiosity-driven follow-up questions were used throughout to elicit richer data [7]. All data were transcribed verbatim and participants given pseudonyms.

### 2.2. Data Analysis 

To identify main patterns in the data without restriction to a preexisting coding scheme, an inductive thematic analysis was conducted on transcripts using a six-phase procedure [10]. Initially, the first and second authors immersed themselves with the transcripts and then generated initial codes. Next, codes were collapsed into potential themes and all data relevant to each potential theme were gathered. Themes were reviewed against transcripts and the entire data set before these were refined and combined into larger themes and sub-themes. This process led to the emergence of 3 main themes and 10 sub-themes, and the initial naming of these. Following a return to the literature on housing and disability, each theme and sub-theme title was amended to reflect this literature. This is not to suggest that the themes or sub-themes were arrived at deductively. Rather, names first given to the themes and sub-themes were refined following later readings of the literature, thereby providing connections with established work rather than reinventing ideas and adding confusion to terminology. Themes and sub-themes were thus generated inductively, each one emergent from the data and representing an identified patterned response or meaning. 

### 2.3. Validity 

The study was guided by a relativist approach to conceptualizing validity in qualitative research [8,11]. This approach does not mean that ‘anything goes’. Rather, it means that criteria for judging the quality of qualitative research are drawn from an ongoing list of characterizing traits as opposed to being applied in a universal manner to all qualitative research [8,11,12]. Here, this list included the following criteria: the worthiness of the topic; the significant contribution of the work; rich rigor, that is, developing a sample appropriate for the purpose of the study and generating data that could provide for meaningful and significant claims; and the coherence of the research, which refers to how well the study coheres in terms of the purpose, methods, and results [8,13]. Participant reflections on our analytical interpretations (or what are sometimes known as member checks) were also utilized, not in an effort to achieve theory-free knowledge, but rather to open up dialogue about the fairness, appropriateness and believability of the results shared [8]. We also each kept a reflexive diary in order to critically reflect on, for example, our prior assumptions held about care homes and SCI, and our ongoing judgments about the data and interpretations of these. As part of a list of characterizing traits for enhancing the quality of our work, the study moreover used an audit trail (*i.e.*, a colleague independently scrutinized data collection and theoretical matters) and aimed for resonance (e.g., naturalistic generalizations) [13]. 

## 3. Results

The analysis process resulted in the development of 3 final themes and 10 subthemes. The main themes were termed damage to quality of life, damage to physical health, and damage to psychological wellbeing. Each theme describes the impact of being in a care home on spinal cord injured adults. Each sub-theme explains the reasons as to why quality of life, health, and wellbeing was damaged. Consistent with conceptualizing the home as a relational and temporal space, the themes and sub-themes are not discrete but overlap in relation to an individual’s contextual circumstances and as life is lived in, through and over time [4]. 

### 3.1. Damage to Quality of Life

Quality of life (QoL) here refers to an individual’s overall perception of and satisfaction with how things are in their life [14]. All the people with SCI consistently expressed that QoL was drastically reduced and damaged when living in the care home. There were several reasons, all attached to living in a care home, for the damage to QoL that the participants talked about and felt they experienced. These are captured in the following sub-themes.

#### 3.1.1. Lack of Independence: Freedom, Control, and Flexibility 

For all the participants, moving into a care home resulted in them losing their freedom, control over most of their daily activities, and flexibility to make personal choices and engage in desired activities [4]. As a result, they felt they no longer lived an independent life whilst living in a care home. For example, participants spoke of having to fit into timetabled meals, leisure activities, and care home staff routines and timetabled meals.

*“For three years in this care home I’ve lived under other people’s rules. You can’t do this, you can’t do that. I’ve no control over my destiny. I like to go to bed when I want, not when someone like staff tell me, I’m not a five-year old. But no, it’s up to them. All my independence has gone since living in here. This place has taken it away. My quality of life as a result has suffered immensely. I’ve no quality of life now, and feel like I’m not even a human being anymore.*”

(Lawrence)

“*It’s easier for them* *(care home staff) to just leave me lying in bed. But I insist every day and tell them,* *“What time are you getting me up?” Sometimes they might say,* *“We can’t get you up today; we’re short staffed”, and then I have to accept that. I’ve no control even over the very basics like getting up in the morning.*”

(Phillip)

#### 3.1.2. Inability to Participate in Community Life

Closely linked to a lack of independence that engendered a low QoL were broader issues concerning the ability to participate in community and civic life [4]. For example, participants discussed that living in a care home severely limited their opportunities to leave this environment, interact in the local community, and participate in social and civic life. In addition to the tightly structured daily schedules, care homes were often extremely distant from the communities the participants once lived and participated in prior to SCI. Moreover, public transport to places that could foster social engagement, such as accessible leisure facilities, pubs/bars, restaurants, libraries, shops, and friend’s houses, was limited or private transport too costly. As the following data testifies, due to the care home environment severely limiting people’s ability to participate in community life QoL was further damaged.

“*I’m stuck in the care home, not because I want to, but because the care home does that to you. It’s like a prison. I miss so many things, and my quality of life now is zero. I can’t get to friends, which I’d love to see, can’t do simple things like shop, or have a drink with a friend. My world has shrunk to this place* *[care home], and with it has gone my life quality. Enjoying life in the community, which is what I hoped for in rehab, is a long way off whilst I’m in here. It’s a prison that has even stopped me seeing friends, family.*”

(Tina)

#### 3.1.3. Inability to Sustain Meaningful Relationships 

For the participants sustaining meaningful relationships included the ability to both give and receive love, care, friendship, companionship and social support in the form of emotional, informational, esteem, and tangible supporting relations. Although not easy, meaningful relationships following SCI were largely maintained whilst in rehabilitation, with some people reporting that relationships with family or friends had even improved. Participants reported that living with loved ones in the same home environment, maintaining social roles as parents and spouses, having a sense of community, and maintaining relationships with friends and/or pets when discharged back into the community was something they deeply looked forward to and perceived vital for their future QoL, health and wellbeing. 

However, the ability to maintain or form new relationships was severely damaged by living in a care home. In some cases, the participants also reported that being in a care home resulted in relationships ending. With the participants living in a care home, and their spouses and children living in their family house they once shared before SCI, relationships were put under great mental and physical strain. Some participants described feeling deeply embarrassed about being in a care home, and therefore instructed their friends, spouse, and young child or children not to come. The lack of activity and stimulation in the care home meant there was nothing to do together or talk about when people visited. Without the ability to drive or with limited transit options, family and friendship relations were also increasingly difficult to maintain. In consequence of the damage to relationships, people felt isolated, insular, and lonely. Their QoL was reduced further.

Not only were relationships damaged and difficult to maintain when in the care home environment, developing new friendships was often perceived as impossible. Each participant described the care home they lived in as a socially sterile environment. There was little meaningful social interaction for the participants. They perceived this was often compounded because elderly people suffering from dementia or another chronic illness largely occupied the care home they lived in. Many of the participants felt deep compassion toward these residents. However, all people with SCI reported that it was extremely difficult—if not impossible—to forge new relationships, share stories, or talk about everyday life with the other residents. Moreover, the participants described that that living with elderly people who suffer from illnesses like dementia impacted negatively on their QoL. For example, the regular “random” shouting from, and the distressed behaviour of, many elderly people caused each participant much distress. The participants also described that their QoL suffered due to elderly care home residents frequently intruding on their personal space during the day or night. This was particularly distressing for young spinal cord injured people.

“*I really struggled to cope in there* *[care home]. My life disintegrated in there, and I felt so hopeful about life toward the end of rehab. I’d broken my neck, but I was up for the challenge. And then, bang, they throw you in a care home. In there, it’s depressing. It’s distressing too I tell you. Let me offer a typical example. At night, they* *[elderly people with dementia] would open the door and come in. I kept telling them not to, but they didn’t respond. I felt helpless to get them to go away because I couldn’t move, and by the time the carers came to take them away, I was already woken up and couldn't get back to sleep. I lay awake, worrying. I’m a young man who was living with old people who had dementia. I felt for them, but it was horrible. Would you send your young son off to live in care home like that for a few years? What quality of life would they have? None I tell you. I had none, and my relationships deteriorated living in the care home; most fell apart. I couldn’t make new friends either. I was thrown into a home with people who were so different to me and who I just couldn’t relate to. My quality of life was zero in the care home. My mental and physical health really suffered too.*”

(Norman)

### 3.2. Damage to Physical Health

Another key theme in the spinal cord injured people’s accounts was that living in a care home has a major negative impact on physical health. Bones were broken and wrong medication dispensed in the care home. Also the participants developed pressure sores, urinary tract infections and autonomic dysreflexia. Such damage to physical health was not caused by the spinal injury per se but rather attributed to major problems within the care home environment. These problems that were key to risking and damaging the physical health of the participants are captured in the following sub-theme.

#### Safety

The care home environment was deemed to be unsafe in terms of the facilities and the competency of attendant care providers. For example, bed mattresses and bathroom facilities in the care homes did not meet the specific needs of the participants. As consequence, despite every personal effort on the part of each participant, it was common for him or her to suffer urinary tract infections, pressure sores, and bacterial infections.

“*I was a good patient in rehab. I learnt how to care for myself in there. But in here* *[the care home], even with my greatest will, all my effort, my health has deteriorated. The facilities are shocking. They might be ok for old people in here, but not a spinal cord injured person. The bed is wrong for me, like the bathroom. Washing is difficult, really difficult and I’ve had infections in here because I can’t wash, or dry myself right, and safely managing my bowels and bladder is always a concern. I’ve had numerous pressure ulcers, and urinary tract infections in here, all because the facilities aren’t right for me.*”

(Owen)

“*I didn’t have the right mattress for me at the care home. I came out of rehab and I’d never had a pressure sore. Within two weeks of getting there* *[care home], I had a grade 4 pressure sore on my heel. By the time I left, I had eight pressure sores. The lack of know-how from staff and the fact I needed certain home equipment, which the care home didn’t have, meant my health suffered. I was lucky I think to come out alive.*”

(Sean)

In addition to the care home facilities, the lack of specialist knowledge and training amongst staff in how to physically care for a spinal cord injured person’s needs, placed the physical health of people with SCI in danger. For example, the participants described countless incidents in which they were incorrectly turned in, or transferred out of, bed by care home staff. When this occurred, pressure sores developed, physical pain could follow, bones could break, and infections and catheterization problems happened. None of this is to say that participants always had bad things to report about care home staff. Many of the participants highlighted cases or singled out certain staff that reflected respectful care relations [4]. For example, participants often recalled a specific staff member who had managed their health needs appropriately, and with dignity and skill. However, reflecting the high staff turnover rates in care homes, staff skilled at caring left regularly. It is also important to note that participants were keen to point out that care home staff frequently wanted to do a good job. They believed that many staff cherished their work and wanted to help. Despite all this, the care home staff were trained to simply look after the needs of the elderly. Staff lacked the knowledge of how to care for the needs of spinal cord injured people. As a result, serious physical health problems often occurred within the care home environment. Further, as the following comments illustrate, the dangers to health that occurred due to living in the care home environment engendered high levels of anger and fear.

“*There are many incidents I could tell you about. A few weeks ago I was being transferred out of bed and the staff put my catheter on the floor, and then went to help me fit it. I’ve told them so many times that I could get an infection from that, but it doesn’t seem to get through. I’ve given up on going to the toilet in here now too. The toilet down the corridor is filled with chairs, and I can’t get down the stairs can I? So what am I supposed to do? Because I’m the only one that would use that toilet, they tell me not use to use the toilet. I’m scared that I’m losing what I learnt in rehab about looking after bladder and bowels now. And this [pointing out arm] was broken in here. Staff tried transferring me with a slideboard, but they didn’t do it properly. Bang. I ended on the floor, my arm broken. They mean well often, but they don’t know how to look after people with a spinal injury. And that is just the half of it. I’ve even been given wrong medication. I could have died. I live in fear in here, I fear for my own life. It’s horrible, gets me so bloody angry too.*”

(Owen)

“*They* *[care home staff] didn’t position me in the chair properly. I was left sat there hunched over for an hour. Not on purpose, but they put my health at risk, and caused me a lot of pain. In here I went too into autonomic dysreflexia. There is a rescue drug called Nifedipine which they’re all supposed to be aware of. I was given paracetamols. What can I say? I just spat them back out. I nearly died. I was so angry.*”

(Sean)

### 3.3. Damage to Psychological Wellbeing

Psychological wellbeing refers to a state of fulfilment and positive engagement with life [15,16]. It denotes a state of positive psychological health and happiness that enables one to live a fully functioning and personally meaningful life. 

In addition to QoL and physical health, living in the care home environment had a profound negative impact upon the psychological wellbeing of people with SCI. There were several conditions that affected their psychological wellbeing. These are as follows.

#### 3.3.1. Restricted Participation in Work and Leisure Time Physical Activity 

Most of the people reported that they wanted to work and engage in physical activity or play disabled sport in order to care for their own physical health as well as psychological wellbeing following SCI. However, the care home environment restricted their ability to compete for work and participate in leisure time physical activities. For example, participants spoke about how the inflexibility of care home schedules, limited independence, a lack of social support, illness, and transportation barriers impeded their ability to vie for work and engage in valued physical activities outside of the care home. Moreover, participants felt that appropriate or enjoyable physical activities for spinal cord injured people were not offered inside a care home.

“*When I knew I wouldn’t walk again I thought,* *“Ok, but there is nothing stopping me being active.” I also knew it would be good for my mental and physical health. So anyhow, when I arrived in the care home I thought* *“Well I may be here but I can still be active, and look after my health, be ready for when I leave”. How wrong I was. More, well, if you look at the activities they do in here* *[the care home] they are all, I say all there aren't many, but what they do is designed for frail, old people…* *The activities didn’t get me out of breath and were boring, very boring. I later found out about a basketball team that plays on a Friday night, and I went to give it go. I really enjoyed it, and felt great. But that stopped pretty quickly because I was relying on staff to take me, and the care home bus. Obviously staff are busy, have other people to see. And also the bus needed to be used for other things. So I had to stop that. Being so far away also meant I couldn’t get a bus back, one that ran later, so here I am, getting fat, sat down or lying down all day, my health being put in danger, even though I want to do something. If I lived in my own house this would not happen. I’d be fit and active again. I’d be mentally healthy, a lot happier, and I’d have some purpose.*”

(Owen)

As these comments illustrate, despite being motivated to care for their health by engaging in physical activity, living in a care home restricted a physically active lifestyle. Given that being active can boost psychological wellbeing for disabled people [16,17,18], the participants were denied access to the benefits that physical activity can bring. In other words, in the care home environment “exercise as a form medicine” was largely restricted, and sedentary behaviour was common. All this not only impacts on people’s wellbeing, but also raises the risk of obesity, heart disease, diabetes, and secondary health conditions that go with SCI like pressure sores [18]. Thus, the damage done to the lives of spinal cord injured people as a result of being in a care home can lead to long-term health and wellbeing problems.

#### 3.3.2. Life on Hold: Lack of Meaning, Self-expression, and a Future 

For people with SCI in this study, living in a care home environment also meant that over time they increasingly lacked a sense of meaning and self-development, self-expression and identity affirmation, and a future to look forward to. As a consequence, not only was psychological wellbeing damaged further. Life was put on hold until a home could be found that would meet their needs. When life is on hold, and a person with SCI is restricted in the activities they can do as a result of living in a care home, the hope and optimism he or she had gained when in rehabilitation about beginning a new life as a disabled person is diminished even further.

“*I feel as though however long I’m in here for, its time lost out of my life. It’s coming up to three years now that I’ve missed out on life. My life is on hold, paused in here… I just can’t get on with my life. Being in here has taken away everything I learned and everything I gained in rehabilitation. My rehabilitation starts when I get out of here. I’m worried though everything I learnt has been lost. It’s madness when you think about it.*”

(Craig)

“*You finish there [rehabilitation] and they tell you it’s possible to have a normal life. But here, it’s not possible. I was full of optimism. That’s gone. A normal life is on hold in here.*”

(Cara)

“*I’m gonna spend the rest of my life in this home, until I end up like these old people. That’s the truth. I’ve no future now. They put all that effort into rehab, and spend loads of money to get me back into society, and then I’m dumped in here. I’ve lost most of what I learnt in rehab. What hope do I have now? I can’t see any.*”

(Arnold)

Not only did the care home environment produce the feeling that life was on hold, that meaning, a purpose and a future was suspended until a new home could be found. Whilst in this environment many of the skills needed to care for new needs that were learnt during rehabilitation eroded over time, thereby putting the health and wellbeing of people in further danger. 

#### 3.3.3. Loneliness

Loneliness can be a positive good and an ontological necessity. However, in this study people’s psychological wellbeing was also damaged due to the intense loneliness that being a body-in-the-world of the care home environment generated. For the participants, loneliness was more than just seclusion or feeling bored. It included a deep felt sense of lost relatedness with others, alienation, and that they do not really belong to themselves anymore.

“*I’ve never experienced anything like it. There is this intense loneliness that is with me all the time. It hangs over me, or engulfs me, taking who I am away. The care home does this to you. There are people about, but I’m lonely in here. The home is destroying me. I’ve been thrown into this prison having broken my neck, and left to rot in loneliness. Do you know what that does you? It makes you even more miserable, sucks life out of you Being in a care home wrecks who you are, totally wrecks you, strips you down to the bone, destroys you, takes away your spirit, your independence, breaks you, just breaks you. It took away who I am. I’m a prisoner here. I’m a prisoner in my body, but that’s not what I’m upset about; that’s accepted. It’s the care home system holds me prisoner.*”

(Harry)

#### 3.3.4. Difficulties with the Re-Housing Process

The participants’ wellbeing was damaged further due to problems with the re-housing process they experienced when in a care home. For example, once in the care home, the participants reported that it was often very difficult for them to obtain information about re-housing from care agencies, social services or council housing authorities. From their position inside a care home, in which they felt they were forgotten about by society, part of this problem was that it was hard to identify and contact a suitable person who could help them.

“*I’m stuck between two boroughs and neither of them want to take charge of my case. I’m writing to my MP at the moment, which really is my last resort. It’s horrible, and if they only knew how miserable and depressed they are making me by passing me on each time and not taking responsibility. It’s not right.*”

(Lawrence)

“*There was no plan to get me out. I had no social worker. Although there were individual people who would have helped if they could, there was no-one who could actually throw a rope down into the cave to help me out—everybody was kind of milling around in there with me. I was trying to get information from all sorts of organisations too, and simply couldn’t get any. Nobody can give you an answer to anything unless they’ve done an assessment based on all the factual details. They just pass the buck somewhere else. I had broken my neck, and all I wanted was some care, and kindness.*”

(Hannah)

After a great deal of effort, frustration, anger, and emotional work, some of the participants did eventually make contact with the appropriate housing authorities and people within it to support the process of re-housing. However, all the participants reported that they felt the housing authorities neglected the urgency of their needs and/or offered them inappropriate housing that felt difficult to reject. For example, when a person was eligible to adapt their existing property through a grant, it was extremely slow to get confirmation of the grant and later obtain it. When the person was not eligible to obtain a grant, or the property could not be adapted, the time taken to find appropriate housing was very lengthy. Moreover, when participants were eventually offered housing the property was often unsuitable to meet their needs as a disabled person. As a result, they felt that their needs were not taken seriously in the process of re-housing. The participants also reported feeling frightened and fearful about having to refuse unsuitable accommodation that was offered to them. All this compounded the damage to their wellbeing.

“*I was offered various places by the local housing association, but those were flats five storeys up, houses with steps leading up to them. The accommodation offered was totally unsuitable for me. And when I go back to them and said* *“This is no good, why am I being offered this?”, they would just turn round and say* *“well, that’s all we’ve got”. It was as though they were just ticking boxes. But, well, I’d get my hopes up about moving out of here, and then they were shattered because the housing wasn’t appropriate. It really felt like no one cared. Devastated me every time, every time.*”

(Craig)

“*This one place I couldn’t even get in the bathroom. But once I accept somewhere, that’ll be it. They won’t give me another option. So even if I think* *“well, I’ll go in there and just make do for a year and see if something else comes up”, that won’t happen. So if I say no again, what will happen then? I’m so scared I’ll end up at the back of a queue and end up in here for even longer. I’m scared. I’m getting more depressed.*”

(Tina)

#### 3.3.5. Depression 

Like many people who sustain a SCI, the participants stated that during their time in rehabilitation they did not suffer from chronic depression or were diagnosed by hospital clinical psychologists as depressed. However, the participants perceived that as a result of living in a care home they became depressed for the first time. They expressed that the combination of feeling that life was on hold, being unable to sustain meaningful social relationships or participate in community life, work or physical activity, isolation, no independence, and persistent difficulties with the re-housing process that were produced as a result of the care home environment led to them feeling sad for weeks, months, and sometimes years. They said they lacked energy, had disturbed sleep, felt helpless and hopeless, had low self-esteem, and were continually unhappy. The interview data also revealed that depression could for several of the participants make it almost impossible to get through daily life.

“*I’ve struggled to admit it, because it’s not really me, like me to admit such things. But I’m depressed. Being in here* *[the care home] has made me depressed. In here I’ve sunk lower than I ever thought humanly possible. I break my neck, feel like my life has ended, but not depressed, gain some hope about my future in rehab, and then have it all swept away when I moved in here* *[the care home]. I’ve never felt as low as I do now. It’s like someone has said ‘Ok, you’ve survived breaking your neck, so now we’re going to throw you into a place to make you even more miserable.*”

(Owen)

“*I felt depressed, I felt incapable of doing everyday things without having the consent of whoever was in charge. I felt like a child, I felt useless, sick, so deeply depressed when in the care home. I could hardly get through each day. A depressing existence, and my depression was down to being in a care home. It continued long after I left, all because I was put in a care home.*”

(Jack)

“*I was so miserable in the care home. I didn’t even know I could cry so much. I was depressed looking back; really depressed. Until I left; I really didn’t know just how deep my depression was in there. I knew I was depressed; but I didn’t realize the extent; just how black my world had turned.*”

(Lisa)

The care home environment therefore was perceived by the participants to have brought about depression. This damage to wellbeing did not go away after the spinal cord injured person left this environment. Such was the devastating impact of being in a care home on psychological wellbeing, it was perceived by those people who had moved out of the care home environment into an appropriate home that depression continued to grip them. That is, living in a care home had an ongoing damaging impact on spinal cord injured people’s wellbeing.

“*The memory of that place still haunts me. I thought I would leave and then it would all be fine, but I’ve got no motivation to do anything anymore—I just stay at home. I still can’t get much sleep at night—I still hear the noises and the shouts. But I can’t even go to the doctor. What will I say,* *“I feel like a hermit”? No, I’d get laughed at. I’m still depressed because of being in the care home.*”

(Norman)

#### 3.3.6. Suicidal Thoughts and Actions 

For most of the participants, the damage done to psychological wellbeing as well as physical health and QoL eventually came together to produce suicidal thoughts and the feeling that life was no longer worth living.

“*Sometimes I think* *“What’s it worth? What’s it all about? Is there an end to this nightmare”?, and then that thought comes into mind—taking the coward’s way out, and that scares me.*”

(Lawrence)

“*I’ve never thought in my life about killing myself. I loved life, and even after my injury, I was going to give it another good go. But living in here, feeling depressed, no quality of life, I lay in that bed and think about killing myself. Sometimes late at night I lay there thinking why don’t* *I just end it.*”

(Owen)

Whilst the majority of the spinal cord injured adults did not act on their suicidal thoughts, several did attempt to take their own lives. For them, the care home environment became too much to deal with—their psychological wellbeing, physical health and QoL had become so damaged—that suicide felt like the only option. They, as the following comments illustrates, would rather be dead than be in the environment of a care home.

“*Just lying in bed all day with no-one to talk to. I stopped taking my medication because I thought* *“I don’t wanna live anymore, I just wanna die.”*”

(Paul)

“*Being in a care home is a horrible experience. I’ve lost all my self-esteem, and my confidence. In here the staff are only used to looking after geriatrics, not paraplegics or tetraplegics. They don’t know how to look after us. And everything I was taught in rehabilitation about looking after myself and living out there with friends and family, in a proper home, has been lost because of being in here. I can’t see any future. There are so many times when I think about ending my life. A few weeks ago, I was lying in bed and thinking* *“What’s the point?” I don’t want to live in an old people’s home. There’s no quality to my life. So I thought I’m going to end it. I managed to move myself in bed, and to shut the ventilator so my lungs would close over. Imagine killing yourself slowly like that. I tried to, but someone brought me back to life again. The staff thought it was an accident. I couldn’t tell them, as I was scared they’d put me in a mental home. And after going to hospital, I was returned back here and they just stuck me in a bigger room. I don’t know what to do. I have no future, no life. I may as well be dead. All I want is an appropriate house to live in.*”

(Arnold)

## 4. Discussion

Despite the large number of people with SCI who now live in an elderly care home, as well as the significance of home environments to human health and wellbeing, there is scant research on how living in a care home environment impacts on the health and wellbeing of people with SCI. This study addressed this gap. The findings reveal that the care home environment severely damages the QoL, physical health, and psychological wellbeing of people with SCI. Analysis of data also uniquely identifies various interwoven reasons as to why the care home environment damaged people’s lives. In so doing, this research makes connections with work that has identified a number of conditions that are necessary for a dignity-enabling home [4]. For example, the participants in this study did not have access to a set of desired conditions that included control and flexibility, community and civic life, self-expression, safety, meaningful relationships, and participation in work or leisure. In this regard, this study offers support to the importance of having access to each of these conditions if a person so desires. It suggests that without these conditions, the care home environment not only can damage the QoL, physical health, and psychological wellbeing of people with SCI. It also fails to enable and promote social dignity. In other words, even when one condition is met in some way (*i.e.*, respectful caring relations), for the participants the lack of freedom, control, and flexibility, safety problems, and inability to participate in community life, sustain meaningful relationships, express oneself in meaningful ways, and participate in work and leisure time physical activity results in a dignity-oppressive home that harms their QoL, health and wellbeing and denies them their fundamental human rights. When seen in such ways, the research connects with both the social relational model of disability [19,20,21] and the human rights model of disability [22,23]. 

Before highlighting these two models, connecting each with the data, and then offering recommendations for policy, it needs stressing that the results of this study may be generalizable or representative in the transferable and naturalistic sense [8,13]. This means that the results resonated with readers with SCI who were living in or had lived in a care home, or readers (e.g., family members) who knew someone in this situation. For example, over 100 people who read the original research report stated that the results directly overlapped with their own experiences of being in a care home and/or produced a vicarious experience of the impact of living in a care home. In these ways, it may be suggested that results meaningfully reverberated with and affected audiences, thereby displaying transferable and naturalistic generalizability.

Building on the social model, the social relational model of disability theorizes disability as a form of social oppression involving the social imposition of restrictions of activity on people with impairments and the socially engendered undermining of their psycho-emotional wellbeing [17,19]. In many respects, the care home could be conceptualized as a relational space that, directly or indirectly, also oppresses the participants. For example, the material environment of the care home and its interactional dynamics restrict activities, such as physical activity and engagement in relationships. The environment may also oppress people with SCI in that it can undermine their QoL, health and wellbeing. For instance, when people are denied appropriate bathroom facilities not only is their safety and physical health put in danger. They also become fearful and frightened. Thus, it is the care home environment that undermines their psycho-emotional wellbeing. In so doing, it fosters disablism, that is, social oppression. 

In addition to violating the collective dignity of people with SCI and oppressing them, care home environments that systematically deny people with SCI opportunities to participate in community and civic life, pursue familial roles, maintain friendships, pursue work and leisure activities, or to enjoy the highest attainable standard of health and wellbeing, violate their fundamental human rights. These rights are captured in the rights based model of disability [22,23,24]. Closely related to the social model, the rights based model states that support in areas like health wellbeing, and housing is not a question of humanity or charity, but instead a basic human right that any person can claim. The model is grounded in the United Nations Convention on the Rights of Persons with Disabilities (UNCRPD) (“the Convention”; United Nations, 2006) [24]. The Convention states that its driving purpose with respect to disabled people is ‘to promote respect for their inherent dignity’ (Article 1, p. 4) [24]. Its provisions require that disabled people ‘have the opportunity to choose their place of residence and where and with whom they live on an equal basis with others and are not obliged to live in a particular living arrangement’, and have access to ‘community support services, including personal assistance necessary to support living and inclusion in the community, and to prevent isolation or segregation from the community’ (Article 19) [24]. Article 23 [24] of the UNCRPD also states that respect for the home and family is a basic human right. Moreover, Article 25 [24] stresses that disabled people have the right to the enjoyment of the highest attainable standard of health without discrimination on the basis of disability. 

The study results provide examples of the many ways that articles 19, 23, and 25 of the UNCRPD, and in turn, how a rights based model of disability have yet to be fully realized in the UK in relation to care homes and people with SCI. While disability laws have resulted in a positive shift for the rights of disabled people, our results highlight how care homes are oppressive and violate the social dignity as well as the rights of people with SCI. Furthermore the UK HM Government Office for Disability Issues, in their *Independent Living Strategy*, indicates that disabled people should have the same level of choice, control and freedom in their daily lives as any other person when it comes to housing (http://odi.dwp.gov.uk/). Our findings provide examples of how this strategy when it comes to housing is not being met by the UK Government or local housing authorities. This failure represents a major economic issue for the health care system and public policy when persons who are able to live in the community occupy costly institutional beds. Although no specific economic data are available in terms of SCI, the financial implications of care home environments in terms of costs to the health system from unnecessary pressure ulcers and other preventable secondary health conditions are huge. The economic costs will also be large as people with SCI often spent years in a care home, with all or most of the costs covered by local government housing authorities. 

In addition to the large economic implications that go with being in a care home, when the UK Government or local housing authorities fail to meet the needs of people with SCI and the *Independent Living Strategy* this failure crucially is an issue of social justice [4]. Framing the care home environment as an issue of social justice, rather than solely as an issue of distribution of scarce resources, helps ensure that the rights of disabled citizens, how are they socially oppressed, and the denial of social dignity is foregrounded in health and public policy debates [4]. A decent minimum living standard, and ethical social practice, needs to honor each person’s humanity and right to a healthy, meaningful, independent, and dignified life in a house that meets their needs. 

With all this in mind, a number of evidence based recommendations are offered. It should be stressed that these recommendations are grounded in the results, which includes data analyzed on how the participants themselves believed that their housing experiences and lives could be improved. When spinal cord injured people are failed by society (including housing authorities and local councils) by being put in a care home, they should be given their own private room in the care home. This space should be respected as their own, and they should be allowed to adapt it according to their needs and tastes. People with SCI need to be consulted regularly on what their needs are, and how these can be met. They need to be treated as the experts of their own bodies, directing their own care. If people with SCI are put in a care home, staff require urgent training in the specific care needs of spinal cord injured people. Currently in the UK staff in care homes do not receive formal certified training in caring for individuals with SCI. Although this may be due to the low professional status given to care home staff in UK society, the high turn-over rates of staff, and/or the economic costs of training staff, this situation needs reversing; staff need formal and certified training. Care homes also need appropriate facilities and equipment to meet the needs of both young and older spinal cord injured people. In addition, speedier processes are urgently needed to enable people with a SCI to obtain grants to move into an adapted home. The longer the process to obtain grants to adapt properties, the more damage is done to spinal cord injured people’s health and wellbeing.

None of these recommendations concerning improving the care home environment are however a viable solution to truly challenging oppression/disablism and promoting QoL, health, wellbeing, social dignity and human rights among SCI people. Instead of damaging the lives of this group of people by placing them in a care home, the most viable solution is more accessible housing. The following recommendations are therefore offered.

First, people with SCI should be discharged from rehabilitation into an adapted property, either in the private or public sector, that meets their housing needs and supports their right to independent living. This should ideally be located close to the individual’s previous home, or in a location of their own choosing. This will enable a more successful transition from rehabilitation into the community as a newly disabled person. It would not be simply a cost-effective solution for meeting the housing needs of people with SCI and their families. A house that meets a disabled person’s needs will help in ensuring that their human rights to health, rehabilitation, adequate living standards, and independent living are protected and maintained. To accomplish the need for more accessible homes, housing rule reforms are also urgently needed. All political parties need to commit to this.

Second, disability friendly social housing that *truly* meets people’s needs should be identified by housing associations and local authorities regularly. These associations and authorities also need to work together to create and maintain an up-to-date accessible housing register so people can be offered, or themselves find truly accessible social housing more easily. 

Third, most often new housing does not meet the needs of people with SCI. Amongst the possible reasons for this are a lack of awareness amongst architects and builders of disabled people’s housing needs, limited disability rights law enforcement, and economic drivers [1]. This situation needs reversing. A significant amount (*i.e.*, a minimum of 10%) of new homes need to be built to full wheelchair accessibility standards (e.g., Lifetime Homes Standards in the UK). This will not only be highly beneficial for many disabled people. Building more accessible new homes will also benefit the increasing ageing population, as they can have similar housing needs to disabled people. Accessible and adapted housing will be an investment that will significantly help the current and future ageing and/disability population. 

## 5. Conclusions

The purpose of this original study was to examine the impact of being in a care home on the lives of people with SCI. Like work on young people in care homes in Australia (http://www.ypinh.org.au/), and perhaps also extending into other populations like those with acquired brain injury or multiple sclerosis, the results reveal that people with SCI are placed in facilities that do not meet their specific needs. In so doing, the care home environment damages people’s QoL, health and wellbeing in the short and long-term. The damage done to the lives of people with SCI as a result of being in a care home cannot continue. Any default position for health or public policy that places spinal cord injured people in a care home means that these policies are damaging the health and wellbeing of people with a SCI. It means that people with SCI will continue to be oppressed, denied social dignity and deprived of their essential human rights. Action and policy change is thus urgently needed. The priority of these changes should be focused on providing more accessible housing that meets the needs of spinal cord injured people so that they can lead fulfilling, independent, and meaningful lives. 
